# Folliculin Regulates Ampk-Dependent Autophagy and Metabolic Stress Survival

**DOI:** 10.1371/journal.pgen.1004273

**Published:** 2014-04-24

**Authors:** Elite Possik, Zahra Jalali, Yann Nouët, Ming Yan, Marie-Claude Gingras, Kathrin Schmeisser, Lorena Panaite, Fanny Dupuy, Dmitri Kharitidi, Laëtitia Chotard, Russell G. Jones, David H. Hall, Arnim Pause

**Affiliations:** 1 Goodman Cancer Research Center, McGill University, Montréal, Québec, Canada; 2 Department of Biochemistry, McGill University, Montréal, Québec, Canada; 3 Department of Physiology, McGill University, Montréal, Québec, Canada; 4 Department of Neuroscience, Albert Einstein College of Medicine, New York, New York, United States of America; University of Washington, United States of America

## Abstract

Dysregulation of AMPK signaling has been implicated in many human diseases, which emphasizes the importance of characterizing AMPK regulators. The tumor suppressor *FLCN*, responsible for the Birt-Hogg Dubé renal neoplasia syndrome (BHD), is an AMPK-binding partner but the genetic and functional links between FLCN and AMPK have not been established. Strikingly, the majority of naturally occurring *FLCN* mutations predisposing to BHD are predicted to produce truncated proteins unable to bind AMPK, pointing to the critical role of this interaction in the tumor suppression mechanism. Here, we demonstrate that FLCN is an evolutionarily conserved negative regulator of AMPK. Using *Caenorhabditis elegans* and mammalian cells, we show that loss of FLCN results in constitutive activation of AMPK which induces autophagy, inhibits apoptosis, improves cellular bioenergetics, and confers resistance to energy-depleting stresses including oxidative stress, heat, anoxia, and serum deprivation. We further show that AMPK activation conferred by FLCN loss is independent of the cellular energy state suggesting that FLCN controls the AMPK energy sensing ability. Together, our data suggest that FLCN is an evolutionarily conserved regulator of AMPK signaling that may act as a tumor suppressor by negatively regulating AMPK function.

## Author Summary

The *FLCN* gene is responsible for the hereditary human tumor disease called Birt-Hogg-Dube syndrome (BHD). Patients that inherit an inactivating mutation in the *FLCN* gene develop lung collapse as well as tumors in the kidney, colon, and skin. It is not clear yet what the exact function of this protein is in the cell or an organism. In this study, we used a simple model organism (the round worm *C. elegans*) to study the function of FLCN. We found that it is involved in the regulation of energy metabolism in the cell. FLCN normally binds and blocks the action of another protein (AMPK), which is involved in the maintenance of energy levels. When energy levels fall, AMPK is activated and drives a recycling pathway called autophagy, where cellular components are recycled producing energy. In the absence of FLCN in worms and mammalian cells, like in tumors of BHD patients, AMPK and autophagy are chronically activated leading to an increased energy level, which makes the cells/organism very resistant to many stresses that would normally kill them, which in the end could lead to progression of tumorigenesis.

## Introduction

Birt-Hogg-Dubé syndrome (BHD) is an autosomal dominant neoplasia disorder that was originally described by Hornstein and Knickenberg in 1975 and by Birt, Hogg, and Dubé in 1977 as a disorder associated with colon polyps and fibrofolliculomas of the skin [1], [2]. Toro et al. recognized in 1999 that BHD patients were also predisposed to develop kidney cancer mostly of the onococytic, chromophobe, or mixed subtype [3]. However, later studies showed a predisposition for all subtypes of kidney cancer including clear cell and papillary subtypes [4]. In addition, BHD confers an increased risk of pulmonary cysts, spontaneous pneumothorax, and cysts of the kidney, pancreas, and liver [3], [5], [6], [7], [8], [9], [10], [11], [12], [13], [14].

The gene responsible for BHD, *FLCN*, was mapped to chromosome 17p11.2 by linkage analysis [15], [16] and identified in 2002 by positional cloning [14]. *FLCN* encodes a novel cytoplasmic 64kDa protein FLCN, which is expressed in most epithelial tissues [17]. BHD patients carry a loss of function germline mutation in one *FLCN* allele and acquire a second hit somatic mutation or loss of heterozygosity (LOH) in the remaining wild-type copy in their renal tumors [18], [19]. In addition, strains of rats, mice, and dogs with a germline mutation in the *Flcn* gene developed spontaneous kidney tumors with a loss of function in the second allele pointing to a tumor suppressor function of FLCN [20], [21], [22], [23]. However, homozygous deletion of *Flcn* resulted in embryonic lethality in these species [24], [25]. Finally, ablation or restoration of *FLCN* in human cancer cells revealed tumor suppressor function in xenograft and soft agar assays [24], [26].

Though the FLCN protein presents no significant homology to any known protein, it is highly conserved from unicellular organisms (yeast) through mammalian species (rodents, dog, humans). Moreover, two 130 kDa folliculin-interacting proteins, FNIP1 and FNIP2 have been identified [27], [28], [29] and implicated in some of the *FLCN* phenotypes in B-cell and stem cell differentiation, and the regulation of apoptosis upon DNA damage [30], [31], [32], [33]. Several studies identified both FLCN and FNIP1/2 as AMPK (5′AMP-activated protein kinase) binding proteins [28], [34], [35], [36]. However, no clear role for FLCN/FNIP1/2 in AMPK function has been described, since both inhibition and stimulation of AMPK have been reported upon loss of function of these genes [32], [37]. Strikingly, the majority of naturally occurring *FLCN* mutations predisposing to BHD were predicted to generate truncated proteins unable to bind AMPK pointing to an essential role of this interaction in the tumor suppressor function. Since we and others have observed that FLCN regulates cellular metabolism [37], [38], [39], we hypothesized that FLCN may regulate cellular energy metabolism through its interaction with AMPK.

AMPK is an evolutionarily conserved master regulator of energy metabolism [40], [41], [42]. When energy levels drop, AMP or ADP bind to the γ regulatory subunit of AMPK and induce an allosteric conformational change [43], [44]. This change leads to the activation of AMPK through phosphorylation of a critical threonine residue (Thr172) in the catalytic subunit and inhibition of its dephosphorylation. When animals and cells encounter stressful environmental conditions leading to lower energy levels, activated AMPK phosphorylates downstream metabolic targets to generate ATP and maintain bioenergetics [40], [41], [42]. For instance, AMPK activates autophagy, a lysosome-dependent degradation process that recycles cytosolic components to generate new cellular components and produce energy [45]. Recently, AMPK was shown to activate autophagy via binding and phosphorylation of the autophagy initiation kinase ULK1, Beclin 1, and Vps34 [46], [47], [48].

Since studies in mammalian cells have led to unclear roles for FLCN in AMPK function, we decided to study the genetic relationship between FLCN and AMPK in the model organism *C. elegans*. FLCN and AMPK are conserved in *C. elegans* and loss-of-function mutants are viable. AMPK activation promotes lifespan extension in *C. elegans* [49], [50], [51], [52] and increases resistance to oxidative and other stresses [51], [52], [53], [54], [55], [56], [57].

Here we show that FLCN controls a distinct evolutionarily conserved energy stress pathway by acting as a negative regulator of AMPK function. Loss of FLCN function led to constitutive AMPK activation, which increased autophagy, resulting in inhibition of apoptosis, higher bioenergetics, thereby enhancing survival to several metabolic stresses. Specifically, we find that the chronic activation of autophagy upon loss of FLCN modifies cellular metabolism, providing an energetic advantage that is sufficient to survive metabolic stresses such as oxidative stress, heat, and anoxia. We confirmed these *C. elegans* results in FLCN- deficient mouse embryo fibroblasts (MEFs) and human cancer cells demonstrating strong conservation of this pathway throughout evolution. Our results demonstrate that FLCN inhibits AMPK and autophagy functions, which may lead to inhibition of tumorigenesis.

## Results

### Loss of *flcn-1* confers resistance to oxidative stress in *C. elegans*

**Figure 1:**
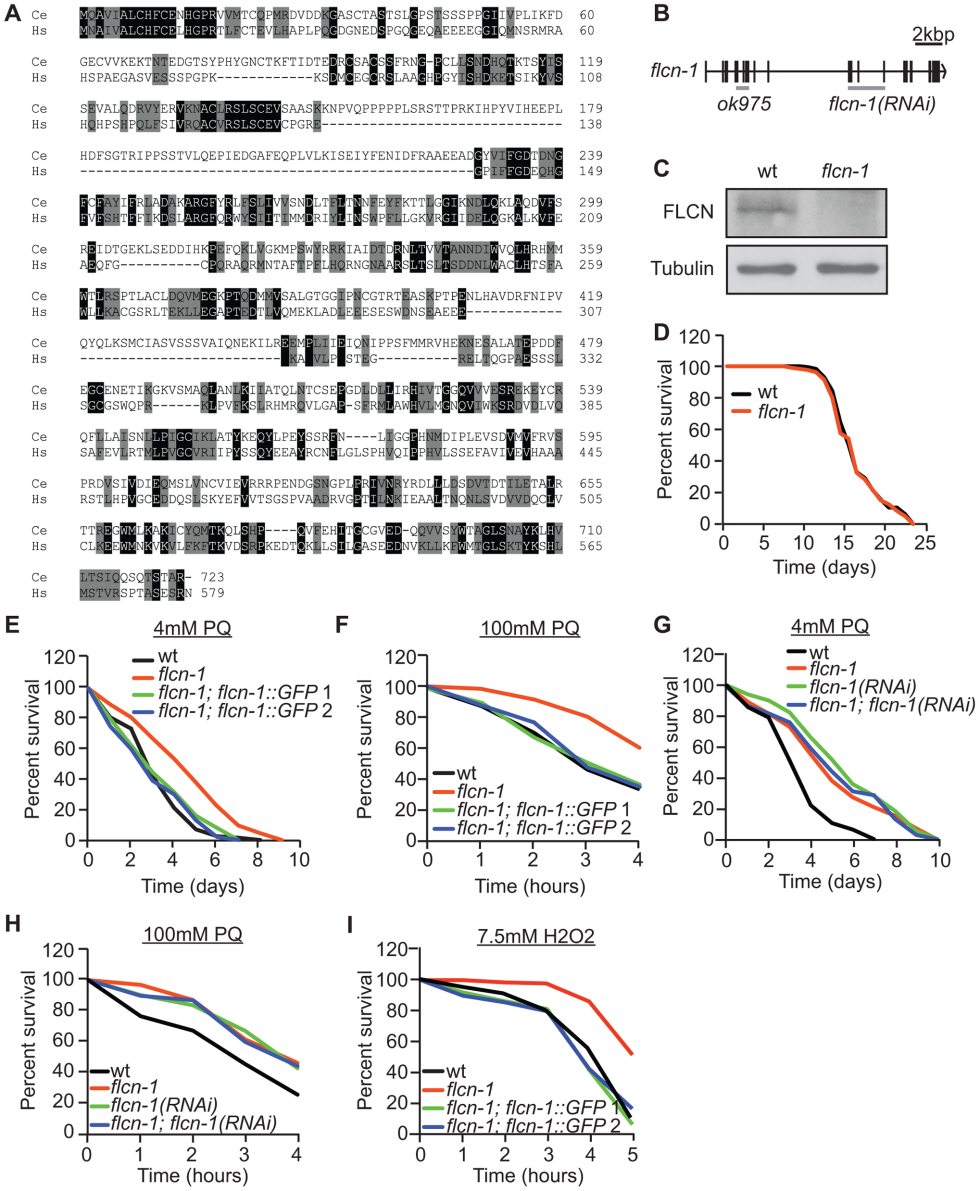
Loss of *flcn-1* confers resistance to oxidative stress in *C. elegans*. (A) Amino acid alignment of the human and *C. elegans* Folliculin sequences (accession numbers: human AF517523, *C. elegans* HE963850). Identical (black) and similar (grey) amino acids are highlighted. (B) Genomic structure of *flcn-1*. The *ok975* mutation and the genomic region targeted by RNAi are indicated. (C) Western blot analysis of FLCN-1 protein levels in wild-type and *flcn-1(ok975)* worm protein lysates. (D) Lifespan of wild-type and *flcn-1(ok975)* nematodes at 20°C (also see Table S1). (E-H) Percent survival of indicated worm strains treated with 4 mM or 100 mM PQ (also see Tables S2, S3). (I) Percent survival of indicated worm strains treated with 7.5 mM H_2_O_2_.

The *FLCN* gene product is highly conserved from *C. elegans* to humans with 28% identity and 50% similarity (Figure 1A). To determine the function of FLCN and whether it genetically interacts with AMPK in *C. elegans*, we used a strain carrying the *flcn-1(ok975)* mutation. The *ok975* mutation is an 817 bp insertion-deletion, predicted to truncate the protein at residue 141 resulting in a null or loss-of-function allele (Figure 1B). In accordance, the *C. elegans* FLCN-1 polyclonal antibody that we developed recognized a gene product at the predicted size in N2 wild-type but not in *flcn-1(ok975)* animals (Figure 1C). Importantly, we did not detect obvious developmental or morphological defects in *flcn-1(ok975)* animals compared to wild-type.

The *C. elegans* AMPK ortholog (*aak-2*; α2 catalytic subunit) modulates longevity and tolerance to stresses including oxidative stress, heat, anoxia, and dietary restriction [49], [50], [51], [52], [54], [56]. Since we did not observe a difference in lifespan between wild-type and *flcn-1(ok975)* animals (Figure 1D and Table S1), we investigated the function of FLCN-1 in stress response by treatment of animals with mild and acute oxidative stress. The *flcn-1(ok975)* mutation conferred an increased resistance to 4 mM and 100 mM paraquat (PQ), a superoxide inducer [58], which could be rescued in two different transgenic lines expressing FLCN-1 (Figures 1E and 1F and Tables S2 and S3). In addition, treatment with *flcn-1* RNAi increased the resistance of wild-type animals to low and high concentrations of PQ but did not further increase the resistance of the *flcn-1(ok975)* mutant animals, supporting that the *ok975* mutation is a loss-of-function allele (Figures 1G and 1H). A similar resistance phenotype was observed upon H_2_O_2_ treatment and was rescued with the two transgenic lines expressing *flcn-1* (Figures 1I and S1A). To exclude the possibility that the changes in stress resistance are not due to effects on lifespan of the animals, assays performed on 4 mM PQ were accompanied with lifespan controls (Table S2). In conclusion, these results demonstrate that loss of FLCN increases resistance to oxidative stress in *C. elegans*.

### Loss of *flcn-1* confers an *aak-2*-dependent resistance to oxidative stress

**Figure 2:**
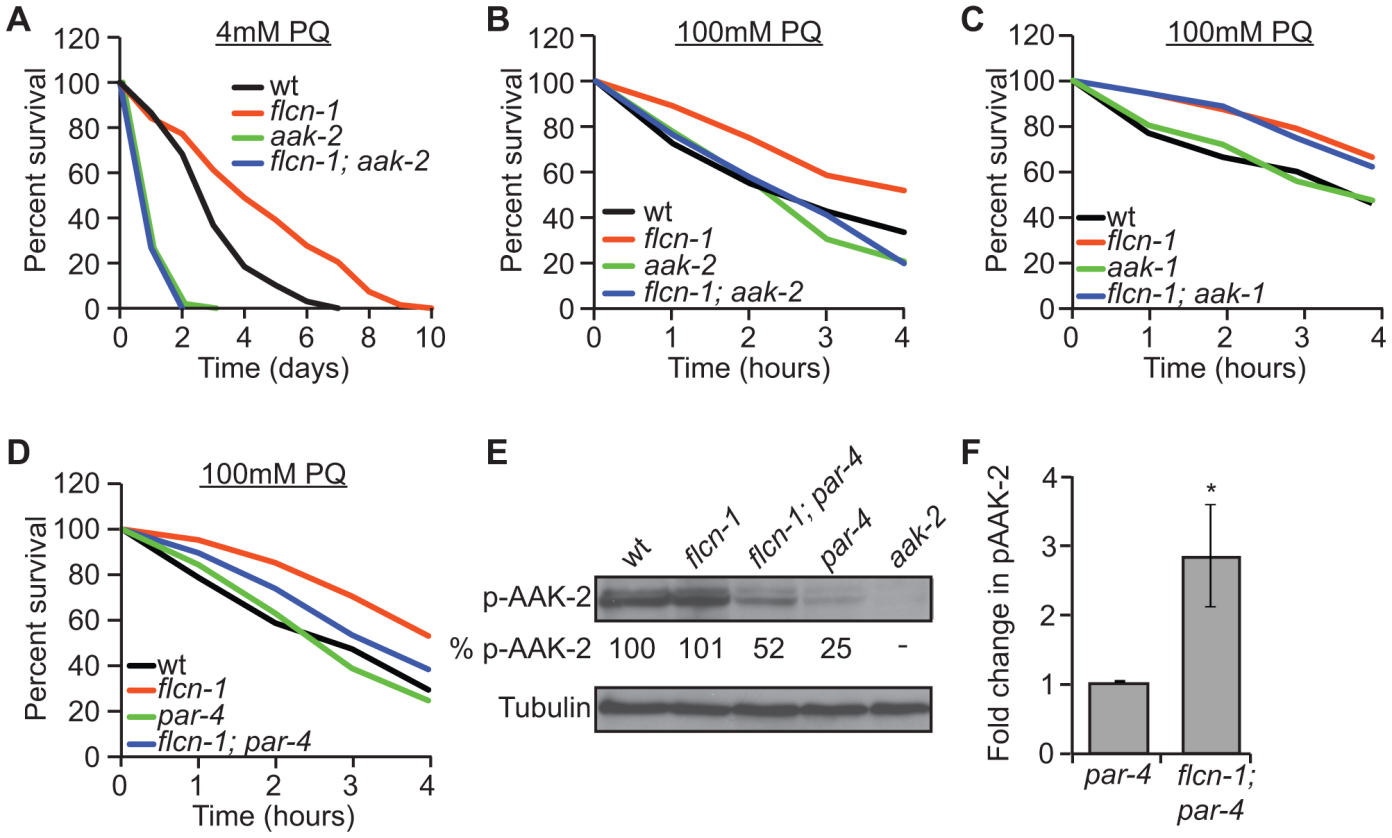
Loss of *flcn-1* confers an *aak-2*-dependent resistance to oxidative stress. (A, B, C, D) Percent survival of indicated worm strains treated with 4 mM or 100 mM PQ. See also Tables S2 and S3. (E) Western blot analysis of pAAK-2 (Thr234) protein levels in indicated worm strains. Levels were normalized to Tubulin. (F) Fold change in pAAK-2 levels in *flcn-1(ok975);par-4(it57)* and *par-4(it57)* animals. Data represent the means ± SEM, n≥3.

Since FLCN binds to AMPK in mammalian cells, we aimed to determine whether *flcn-1* and *aak-2* interact genetically in *C. elegans*. Similarly to published results [50], [51], [52], [54], *aak-2(ok524)* mutant animals were more sensitive to PQ stress compared to wild-type (Figures 2A and 2B and Tables S2 and S3). Strikingly, *flcn-1(ok975); aak-2(ok524)* double mutant animals displayed reduced survival upon treatment with 4 mM PQ (Figure 2A and Table S2) and 100 mM PQ (Figure 2B and Table S3), similarly to *aak-2(ok524)* single mutants, indicating that *aak*-2*(ok524)* is required for the *flcn-1(ok975)* phenotype. The *C. elegans* AMPKα1 homolog (AAK-1) was previously shown to be dispensable for oxidative stress resistance [54]. Accordingly, the *aak-1(tm1944)* mutation did not abolish the increased survival of *flcn-1(ok975)* mutants to PQ (Figure 2C and Table S3). To further test whether the increased survival of *flcn-1(ok975)* mutants was also dependent on PAR-4, the *C. elegans* ortholog of LKB1 and major upstream kinase of AMPK [40], [54], we measured survival to PQ upon *par-4* loss. Interestingly, *par-4(it57)*, a strong loss of function allele, only partially suppressed the *flcn-1(ok975)* survival phenotype, leading to a significant increase in the resistance of *flcn-1(ok975); par-4(it57)* animals to PQ compared to *par-4(it57)* animals alone, suggesting that additional inputs might activate AAK-2 to mediate survival (Figure 2D and Table S3).

Based on these results, we anticipated that loss of *flcn-1* might lead to a constitutive activation of AAK-2 since the observed *flcn-1* loss of function phenotype is similar to the reported AAK-2 overexpression in terms of oxidative stress resistance [49], [52]. Although we did not observe an increased abundance of phospho-AAK-2 at residue Thr234 (corresponding to Thr172 in human AMPKα) in *flcn-1(ok975)* animals compared to wild-type, our data demonstrate a significant increase in phospho-AAK-2 levels in *flcn-1(ok975); par-4(it57)* double mutants compared to *par-4(it57)* animals (Figure 2E and 2F). This is consistent with the observation that *par-4* is not fully required for the stress resistance phenotype (Figure 2D and Table S3). To exclude the possibility that the increased phosphorylation of AAK-2 is due to a *flcn-1*-dependent increase in total AAK levels, we measured the mRNA expression of *aak-1* and *aak-2* in wild-type and *flcn-1(ok975)* animals and did not observe a significant difference (Figure S1B). Interestingly, the residues flanking the mammalian AMPKα (Thr172) are conserved in AAK-2 (Thr234), but are different in AAK-1 and therefore, AAK-1 is unlikely to be detected by the antibody used (Figures 2E, lane 5). The increased phosphorylation of AAK-2 only in *par-4(it57)* mutants can be explained by the fact that PAR-4 is the major kinase that phosphorylates AAK-2 in certain tissues of the animal or cellular sub-compartments, which would mask the FLCN-dependent phosphorylation signal on AAK-2 induced by other upstream kinases [54]. Taken together, these results imply that *flcn-1* negatively regulates *aak-2* in *C. elegans*, and that loss of *flcn-1* confers an *aak-2*-dependent resistance to oxidative stress.

### Resistance to oxidative stress in the absence of *flcn-1* is not dependent on classical ROS detoxification pathways

The insulin/IGF-1-like (DAF-2)/FOXO3a (DAF-16) and target of rapamycin (TOR) signaling pathways are known to control lifespan and stress response in *C. elegans* and other organisms and have been linked to AMPK signaling [50], [52], [59], [60], [61]. While *daf-2(e1370)* mutants exhibited an increased survival to PQ compared to wild-type animals, the *flcn-1* mutation further increased the resistance of *daf-2(e1370)* animals (Figure S2A and Table S3). Consistently, *daf-16(mu86)* slightly reduced but did not suppress the resistance of the *flcn-1(ok975)* animals to PQ (Figure S2B and Table S3). Moreover, we found that the PQ resistance of the *flcn-1(ok975)* animals treated with TOR *(let-363)* RNAi was significantly higher than wild-type animals fed with the same RNAi (Table S3).

Transcriptional upregulation of ROS detoxification enzymes prior to stress could explain the increased survival of *flcn-1(ok975)* [62]. We did not observe a significant increase in the gene expression of superoxide dismutases (*sod-1, sod-2, sod-3, sod-4*, and *sod-5*) or catalases (*ctl-1, ctl-2*, and *ctl-3*), in *flcn-1(ok975)* mutants when compared to wild-type (Figure S2C). Furthermore, we quantified the oxidative damage to protein and DNA. Levels of protein carbonylation and DNA damage were equal in both wild-type and *flcn-1(ok975)* animals under basal conditions and were similarly induced after PQ treatment (Figures S2D and S2E). Taken together, these findings suggest that the increased survival of *flcn-1(ok975)* mutant to PQ may not be dependent on classical oxidative stress resistance mechanisms. A likely mechanism of survival upon loss of *flcn-1* on PQ might involve inhibition of apoptosis.

### Increased autophagy upon loss of *flcn-1* confers resistance to oxidative stress

**Figure 3:**
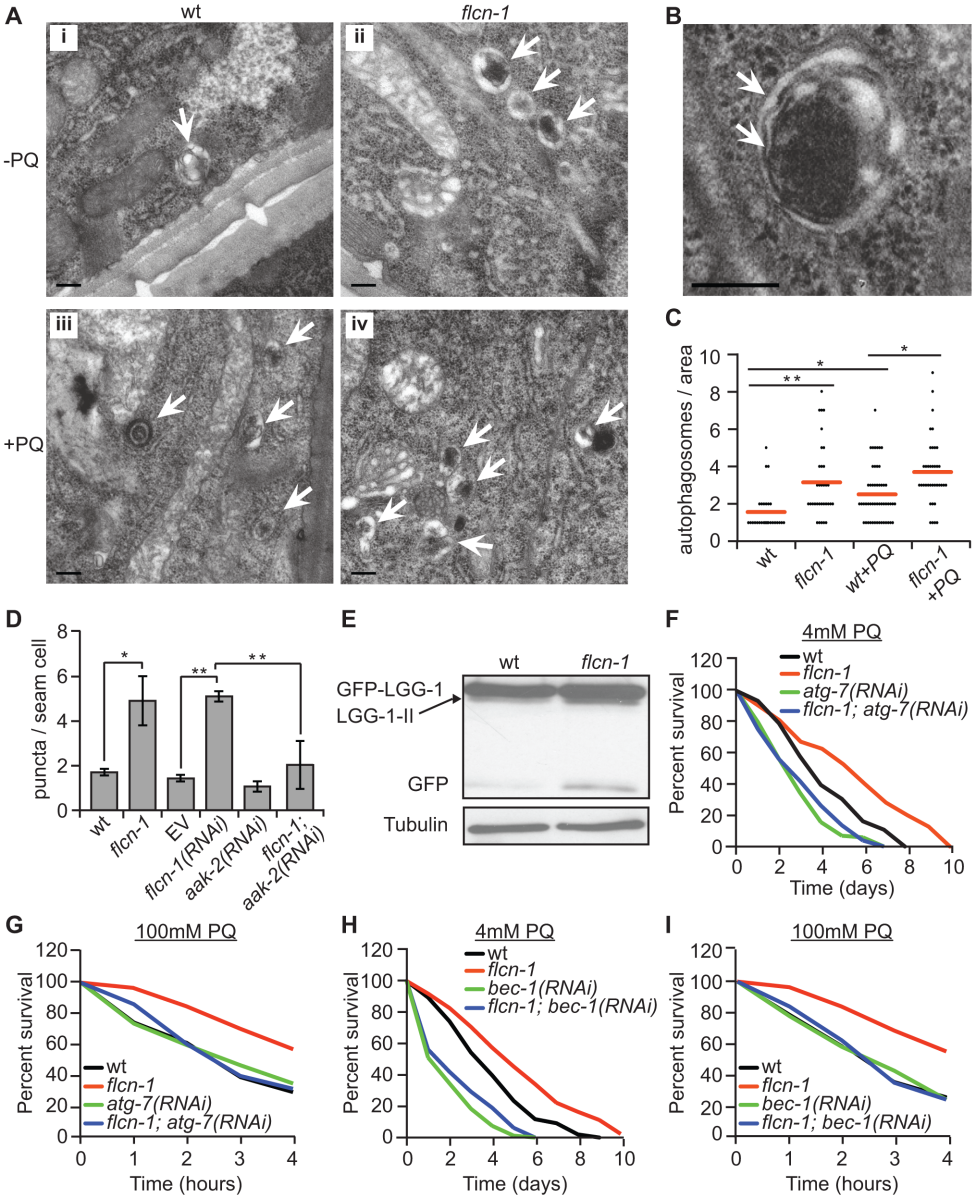
Loss of *flcn-1* activates autophagy resulting in oxidative stress resistance in *C. elegans*. (A and B) Representative electron micrographs from longitudinal sections of the hypodermis in indicated nematodes strains. Arrows represent autophagic vacuoles (A) and autophagosomal membranes (B). Scale bars: 0.2 µm (A and B). (C) Quantification of the autophagic events observed in defined surface area of 4.25 um^2^ of electron micrographs taken from at least 5 animals. Red lines represent the mean of autophagosome numbers per area indicated strains and treatment condition. (D) Number of GFP::LGG-1 positive autophagosome puncta in the seam cells of the indicated worm strains. (E) Western blot analysis of the GFP::LGG-1 cleavage profile (LGG-1-II, GFP) in worm protein extracts. (F, G, H, I). Percent survival of indicated strains upon 4 mM (F and H) or 100 mM (G and I) PQ. Data represent the means ± SEM, n≥3. Also see Tables S2 and S3.

Autophagy has been shown to mediate resistance to oxidative stress across evolution without a clear mechanistic explanation [63]. To investigate whether the increased oxidative stress resistance of *flcn-1(ok975)* mutants was due to autophagy, we measured autophagy levels using several methods. Using electron microscopy, we noticed the frequent appearance of autophagic vacuoles (Figure 3B) in *flcn-1(ok975)* mutants at the basal level compared to wild-type animals which increased under PQ treatment (Figures 3A and 3C). To confirm this result, we used a reporter strain that carries the integrated transgene expressing GFP::LGG-1 (LC3 ortholog). LC3 localizes to pre-autophagosomal and autophagosomal membranes, and GFP-positive puncta are thought to represent autophagosomal structures in this strain [64], [65], [66]. To exclude effects on the transgene expression, we determined the mRNA levels of LGG-1 in wild-type and *flcn-1(ok975)* animals and in the GFP::LGG-1 and *flcn-1*; GFP:: LGG-1 transgenic lines. In both cases, the transcript levels of LGG-1 were not significantly different between wild-type and *flcn-1(ok975)* animals demonstrating equal expression (Figures S3A and S3B). Importantly, we observed a significant increase in the number of GFP-LGG-1 positive puncta in *flcn-1(ok975)* mutants compared to wild-type animals under basal conditions (Figure 3D). Consistently, treatment of GFP::LGG-1 animals with *flcn-1* RNAi increased the number of GFP-LGG-1 puncta (Figure 3D). Previous studies in yeast, *C. elegans*, and mammalian cells have demonstrated that LGG-1-II (or LC3-II) is degraded inside the autolysosomes, and that the GFP fragment is resistant to degradation and accumulates when autophagy is induced [67], [68], [69], [70]. Therefore, we performed western blot analysis on wild-type and *flcn-1* protein extracts to assess the level and cleavage of GFP-LGG-1. Importantly, western blot analysis showed that both cleaved LGG-1-II and released GFP were increased in *flcn-1(ok975)* mutants, indicating higher autophagic activity (Figure 3E).

AMPK has recently been shown to directly induce autophagy in mammals via phosphorylation of autophagy proteins including ULK-1, VPS-34 and BEC-1 [46], [47], [48]. Moreover, loss of *aak-2* reduced autophagy in *daf-2* mutant animals, while *aak-2* overexpression induced autophagy [46]. Based on these results, we questioned whether the increased autophagy in *flcn-1(ok975)* animals depends on *aak-2*. Importantly, RNAi treatment against *aak-2* significantly reduced the number of puncta in *flcn-1(ok975)* mutants, demonstrating an *aak-2*-dependent mechanism (Figure 3D).

Inhibition of autophagy genes in *C. elegans* reduced survival to certain stresses such as anoxia, starvation and pathogens [66], [71], [72]. However, the requirement of autophagy genes in resistance to oxidative stress was not previously reported. We aimed to determine whether the increased survival of *flcn-1(ok975)* animals to oxidative stress was dependent on autophagy. Strikingly, inhibition of the essential authophagy genes *atg-7* and *bec-1* using RNAi markedly abolished the resistance of *flcn-1(ok975)* to PQ (Figures 3F–3I and Tables S2 and S3). Taken together, these results demonstrate that loss of *flcn-1* induces autophagy, which is required for *flcn-1*-mediated stress resistance.

### Autophagy increases energy levels upon loss of *flcn-1*

**Figure 4:**
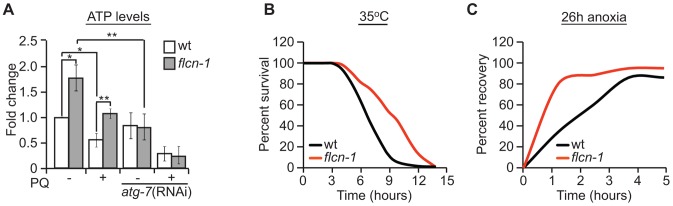
Loss of FLCN stimulates cellular energy production and resistance to energy stress. (A) Relative ATP levels measured in the indicated worm strains treated with or without PQ. (B) Percent survival of wild-type and *flcn-1(ok975)* nematodes upon heat stress (35°C). (C) Recovery rate of wild-type and *flcn-1(ok975)* strains after 26 hours anoxic injury. See Tables S4 and S5. Data represent the mean ± SEM, n≥3.

Autophagy is a process that generates catabolic substrates for mitochondrial ATP production and allows cellular macromolecules to be recycled. Since we did not observe a difference in oxidative damage between wild-type and *flcn-1(ok975)* mutant, and since PQ is known to severely decrease ATP levels by inhibiting oxidative phosphorylation [73], [74], [75], we wondered if *flcn-1* is mediating an increased resistance to energy stress by employing autophagy as a source of energy. To test this hypothesis, we measured ATP levels prior and after 13 hours of 10 mM PQ treatment. Strikingly, we found that *flcn-1* mutant animals have higher levels of ATP before PQ treatment compared to wild-type (Figure 4A). As expected, PQ treatment decreased the ATP levels in both wild-type and *flcn-1*, yet ATP levels in *flcn-1(ok975)* nematodes remained higher than wild-type. Importantly, *flcn-1(ok975)* mutants treated with PQ exhibited equal amounts of ATP when compared to the non-treated wild-type animals (Figure 4A). To test if the increased energy in *flcn-1(ok975)* mutants is dependent on autophagy, we treated the wild-type and *flcn-1* nematodes with *atg-7* RNAi and measured ATP levels. Strikingly, downregulation of autophagy completely suppressed the increased ATP levels in *flcn-1(ok975)* mutants in presence or absence of PQ (Figure 4A).

To further confirm that loss of *flcn-1* confers resistance to low energy levels, we measured the resistance of wild-type and *flcn-1(ok975)* nematodes to heat stress and anoxia, both of which are known to result in a strong depletion of energy [76]. Accordingly, when exposed to 35°C, the mean survival of *flcn-1(ok975)* animals was significantly higher compared to wild-type (Figure 4B and Table S4). In addition, the recovery rates after a 26 hours anoxic injury were faster in *flcn-1(ok975)* compared to wild-type (Figure 4C and Table S5). In conclusion, our data describe a novel mechanism for AAK-2-dependent resistance to oxidative stress, which depends on maintenance of energy homeostasis via autophagy.

### Loss of *flcn* protects against cell death and increases survival to stress

The interplay between autophagy and apoptosis determines the decision between life and death which is very important for the genetic integrity of the cell [77]. The activation of autophagy has been shown to protect against cell death in *C. elegans* and mammals [72], [77], [78], [79]. To see whether *flcn-1* controls apoptosis in animals, we determined the number of apoptotic cell corpses in the gonad arms of wild-type and *flcn-1* animals upon PQ treatment. As expected, we found that PQ treatment significantly increased the number of apoptotic corpses in wild-type animals (Figure S4A). However, the increase in *flcn-1* was much lower suggesting that loss of *flcn-1* protects against cell death. To determine whether the decreased cell death in *flcn-1* nematodes depends on the activation of autophagy, we pretreated the wild-type and *flcn-1(ok975)* nematodes with *atg-7* RNAi and then measured the number of apoptotic corpses upon PQ treatment. Importantly, the inhibition of the autophagy gene *atg-7* increased the number of apoptotic corpses, up to the same level, in both wild-type and *flcn-1* suggesting that the FLCN-1-dependent activation of autophagy protects against cell death (Figure S4A).

Importantly, the apoptotic pathway is conserved from animals to mammals. When cells are destined to die, the BH3 only protein EGL-1 binds and inhibits the BCL-2 homolog CED-9, which activates the caspase CED-3 and leads to death [80]. Therefore, we treated wild-type and *flcn-1(ok975)* animals with *egl-1* and *ced-3* RNAi and assessed their survival to 100 mM PQ. Importantly, downregulation of *egl-1* or *ced-3* using RNAi increased the resistance of wild-type animals which was not observed in *flcn-1(ok975 animals* suggesting that the inhibition of the apoptotic pathway leads to the increased resistance (Figures S4B–S4D and Table S3). Consistently, treatment of wild-type and *flcn-1(ok975)* animals with *ced-9* RNAi abolished the survival of *flcn-1* animals (Figure S4 and Table S3). Importantly treatment of *aak*-2*(ok524)* animals with *ced-3* RNAi did not increase their resistance to PQ suggesting that this phenotype depends on AAK-2 (Table S3). The increased stress response by inhibition of apoptosis has been recently reported [81]. Although it is not clear whether the apoptosis inhibition in the gonad arms delays organismal death in *C. elegans* or whether apoptotic genes acquire non-apoptotic functions, our data suggest an AMPK-dependent involvement of the apoptotic pathway in the increased survival of *flcn-1* animals to PQ stress (Table S3).

### The FLCN-dependent regulation of AMPK, autophagy, apoptosis, and metabolic stress survival is evolutionarily conserved

**Figure 5:**
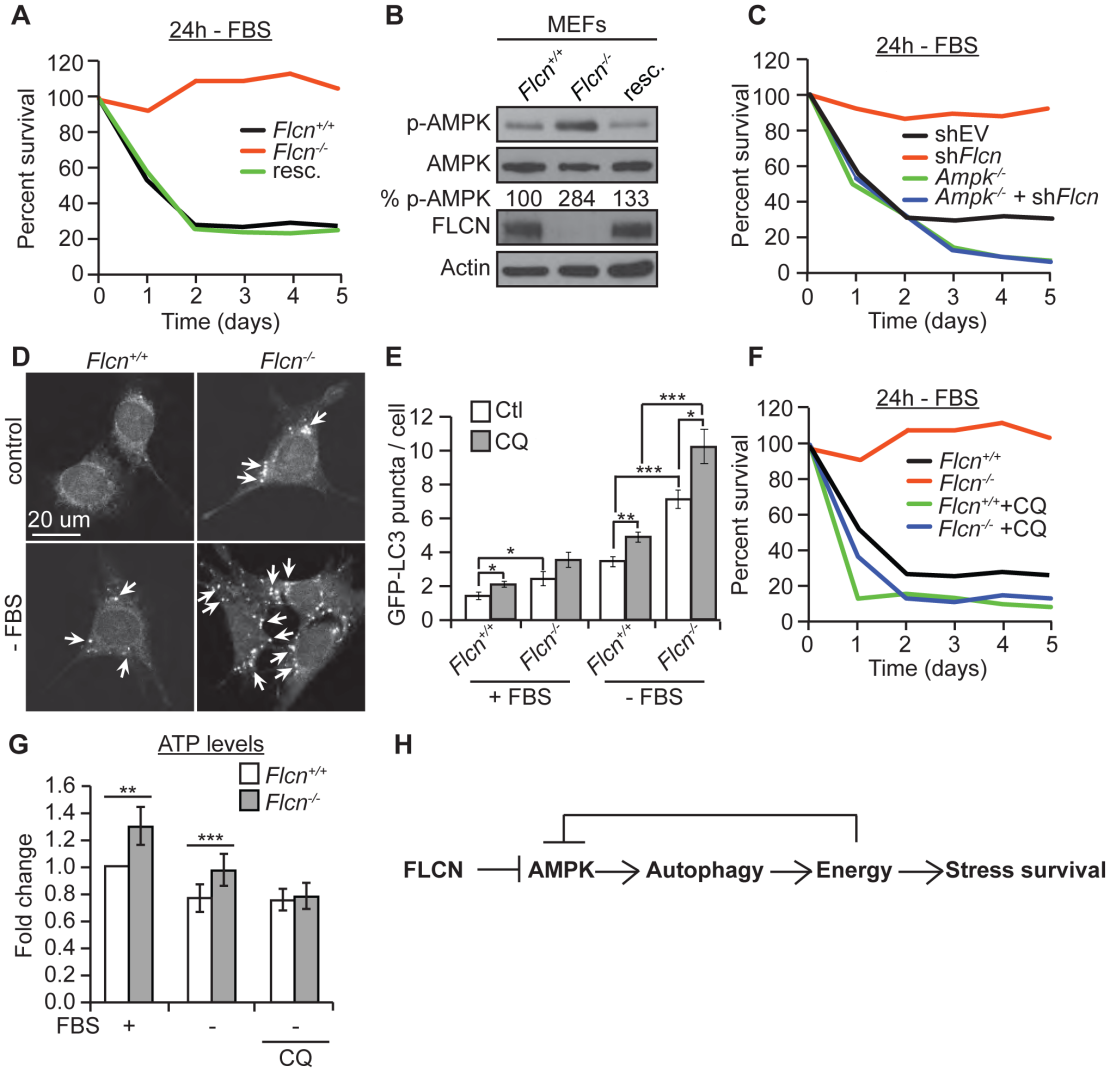
The FLCN-dependent regulation of AMPK, autophagy, and metabolic stress survival is evolutionarily conserved. (A) Percent survival of wild-type, *Flcn* ^−/−^ and FLCN-rescued MEFs (resc.) upon serum starvation (-FBS). (B) Western blot analysis of pAMPK (Thr172) and AMPK protein levels in indicated MEFs lines. (C) Percent survival of the indicated MEF cell lines upon serum starvation. Data represent the means ± SEM, n≥3. (D and E) Representative immunofluorescence pictures (D) and quantification (E) of LC3 positive GFP puncta (arrows) in wild-type or *Flcn* ^−/−^ MEFs under basal or 24 hours serum starvation conditions (-FBS). When indicated, cells were pretreated with chloroquine (CQ) 12 hours prior to serum starvation, N>200 cells for every trial. (F) Percent survival of indicated cell lines upon serum starvation, treated with or without 10 µM CQ. Data represent the mean ± SEM, n≥3. (G) Relative ATP levels measured in the indicated MEFs lines, pre-treated with or without 10 µM CQ prior to serum starvation. (H) Graphical model that summarizes findings of this study.

To test whether the FLCN functions that we identified in *C. elegans* are evolutionarily conserved, we used wild-type (*Flcn^+/+^*) and knockout (*Flcn* ^−/−^) MEFs. First, we examined the cellular resistance to serum starvation (-FBS), which reduces energy levels and induces oxidative stress in a physiological manner (Figure 5A) [58]. *Flcn* ^−/−^ MEFs were unaffected by serum starvation, as demonstrated by a significant maintenance of cell survival after 4 days of serum starvation, which eliminated almost 80% of wild-type cells. Rescue of wild-type FLCN expression (resc.) reverted this protective phenotype (Figure 5A). Consistent with these data, *Flcn^−/−^* MEFs were more resistant to 2 mM H_2_O_2_ treatment compared to wild-type (Figure S5A).

Moreover, in accordance with the *C. elegans* results, phospho-AMPK levels were increased upon loss of *Flcn* in MEFs, which could be rescued by expression of wild-type *Flcn*, suggesting that FLCN acts a negative upstream regulator of AMPK (Figures 5B and S2B). Additionally, down regulation of *Flcn* by shRNA in MEFs lacking AMPKα (*Ampk^−/−^*) did not increase resistance to serum starvation suggesting that the increased resistance to starvation-induced stress depends on AMPK (Figures 5C and S5B).

Next, we asked whether the AMPK activation in *Flcn^−/−^* MEFs could be further activated. Importantly, phosphorylation levels of AMPK and its target ACC were maximal in *Flcn^−/−^* cells and did not further increase upon serum starvation (Figure S5C). Similarly, treatment with AICAR (5-amino-1-β-D-ribofuranosyl-imidazole-4-carboxamide), an AMP analogue, increased AMPK activation in wild-type as marked by elevated pACC levels but not in *Flcn^−/−^*MEFs (Figure S5C). These results demonstrate that loss of FLCN leads to maximal AMPK activation, which appears uncoupled from its energy sensing function.

Moreover, we wondered whether loss of FLCN also increases autophagy in MEFs similarly to the results obtained in *C. elegans*. Importantly, *Flcn^−/−^* MEFs displayed an increased number of autophagosomes at the basal level and under serum starvation conditions when compared to wild-type cells as determined by the GFP-LC3 reporter (Figures 5D and 5E). To validate these results, we used a GFP-mCherry/LC3 reporter [82]. Upon physiological pH in newly formed autophagosomes or when autophagy is impaired, both GFP and mCherry colocalize in puncta whereas upon lysosomal fusion and acidification, the GFP signal is lost and only mCherry is detected. As expected, the number of mCherry puncta was increased in *Flcn^−/−^* MEFs, pointing to a normal lysosomal acidification and completion of autophagy (Figure S6A). In addition, chloroquine (CQ) treatment, which inhibits the acidification of autolysosomes, further increased the number of autophagosomes in *Flcn^−/−^* MEFs suggesting that the autophagy process is not impaired (Figures 5E and S6A). We also measured the rate of conversion of LC3I to LC3II by western blot analysis. The ratio of LC3II to LC3I was increased in *Flcn^−/−^* MEFs at the basal level and was reverted by FLCN rescue (Figure S6B). In agreement with the heightened autophagy, we observed an increase in the activating AMPK-dependent phosphorylation site at the autophagy initiating kinase ULK1 (Figure S5C). To determine whether the increased resistance to serum starvation was due to increased autophagy as we observed in *C. elegans*, we inhibited autophagy using CQ or *Bec-1* shRNA. Inhibition of autophagy strongly suppressed the survival advantage upon serum starvation in *Flcn^−/−^* MEFs (Figures 5F and S6C). Finally, we measured apoptosis in response to serum starvation and inhibition of autophagy in MEFs (Figure S7D). Apoptosis was strongly increased in wild-type MEFs and suppressed in *Flcn^−/−^* MEFs upon serum starvation. Furthermore, inhibition of autophagy using CQ abolished the suppression of apoptosis in *Flcn^−/−^* MEFs. In conclusion, these results correspond well with the data we obtained in *C. elegans*.

Next, we determined whether the chronic activation of autophagy is leading to an energy surplus, which is required for the stress resistance phenotype conferred by loss of FLCN. Similarly to what we found in *C. elegans*, *Flcn* ^−/−^ MEFs displayed increased ATP levels under basal conditions (Figure 5G). Serum starvation decreased ATP levels in wild-type MEFs, while *Flcn^−/−^* MEFs maintained ATP levels at wild-type levels (Figure 5G). Importantly, inhibition of autophagy with CQ abolished the increase in ATP in *Flcn* ^−/−^ MEFs (Figure 5G). The ATP, ADP, and AMP levels as well as phospho-creatine, a short term energy reserve, were all increased at the basal level in *Flcn^−/−^* cells as compared to wild-type, suggesting a general increase in cellular metabolism and energy storage (Figures S7A and S7C). Importantly, serum starvation decreased energy levels in *Flcn^−/−^* cells down to wild-type basal levels (Figures S7A and S7C).

Normally, AMPK is activated upon energy deprivation (increased ADP/ATP and AMP/ATP ratios), which is inconsistent with the increased basal ATP levels conferred by loss of FLCN [40], [42]. To understand this discrepancy, we determined the adenylate energy charge of wild-type and *Flcn^−/−^* MEFs. The adenylate energy charge, which is expressed by the ratio [ATP] + 0.5[ADP]/[ATP]+[ADP]+[AMP], was proposed as a convenient indicator of the cellular energy status [83]. Under normal conditions, ATP levels were increased in *Flcn* ^−/−^ MEFs but the total energy charge was comparable to wild-type (Figure S7B). However, serum starvation significantly reduced the energy charge in wild-type MEFs but not in *Flcn^−/−^* MEFs, suggesting that *Flcn^−/−^* cells derive their energy from an intracellular source of ATP [83] (Figure S7B).

To determine whether loss of FLCN in human cancer cells also conferred an advantage in energy homeostasis, we used the follicular thyroid carcinoma cells FTC-133 lacking FLCN expression, which we rescued for FLCN using stable transfection. First, we confirmed the findings that we obtained with wild-type and *Flcn^−/−^* MEFs. As expected, loss of FLCN conferred an increased phosphorylation of AMPK as well as a higher LC3 cleavage demonstrating that AMPK activation and autophagy in FLCN-deficient FTC cells is elevated compared to rescued cells (Figure S8A). Next, we aimed to determine whether the increased autophagy promoted by loss of FLCN heightens ATP levels at normal conditions in FTC cells. Similarly to what we observed in MEFs, loss of FLCN requires autophagy to increase ATP levels (Figure S8B). To determine whether autophagy contributes to anchorage-independent growth conferred by loss of FLCN, we performed soft agar assays in the presence or absence of autophagy inhibition using *atg7* shRNAs. As expected, loss FLCN significantly increased the number of colonies growing in soft agar in an autophagy-dependent manner (Figures S8C and S8D). Taken together, these data demonstrate that loss of FLCN leads to an autophagy-dependent increase in ATP levels enabling FLCN-deficient animals/cells to resist metabolic stresses, which could constitute a tumor suppression mechanism.

## Discussion

Maintenance of cellular bioenergetics and management of oxidative stress are essential for life. Here we highlight the discovery of an evolutionarily conserved signal transduction pathway mediated by the tumor suppressor FLCN and AMPK that is essential for resistance to metabolic stress. Loss of FLCN in *C. elegans* and mammalian cells leads to constitutive activation of AAK/AMPK, which in turn increases autophagy. Chronic activation of autophagy leads to increased ATP production and confers resistance to energy depleting stresses by inhibition of apoptosis. Together, our data identify FLCN as a key regulator of stress resistance and metabolism through negative regulation of AMPK.

Several questions arise from these results. First, how is AAK-2 being activated in *flcn-1(ok975)* animals upon loss of PAR-4? Several upstream AMPK kinases other than LKB1 have been identified in mammalian cells, have been shown to affect AMPK activity [40]. Although these kinases have not been linked to AAK-2 in *C. elegans*, our data showed a significant basal phosphorylation of AAK-2 in *par-4(it57)* mutant animals. This is in agreement with a recently published study showing that starvation and mitochondrial poisons increased phospho-AAK-2 levels in *par-4(it57)* mutant animals, and that the starvation-induced *aak-2* phenotypes were partially dependent on PAR-4 [84]. The fact that the AMPK signaling pathway is evolutionarily conserved suggests that AMPK upstream kinases other than PAR-4 are likely to exist in *C. elegans*. For instance, Pak1/Camkk-beta was first identified in yeast as Snf-1/AMPK-activating kinase and was proven later to act upstream of AMPK in mammalian systems [85], [86], [87]. Very recently, CAMKII overexpression was shown to increase lifespan in *C. elegans*, although the link to AAK-2 was not investigated [88].Together, our data demonstrate that FLCN-1-dependent regulation of AAK-2 mediates an important novel pathway for stress resistance. Interestingly, this pathway is distinct from previously described AAK-2-mediated oxidative stress resistance mechanisms that involve ROS detoxification [51], [52], [89].

Another unexpected finding is that loss of *flcn-1* did not modulate *C. elegans* longevity under normal growth conditions. The observed increased AAK-2 activation upon loss of *flcn-1* is masked by PAR-4-dependent phosphorylation of AAK-2. General overexpression of AMPK extends lifespan and increase stress resistance [46], [49], [50], [51], [52], [53], [56], [90]. Our data suggest that the signaling cascade downstream of FLCN-1/AAK-2 is different from the AAK-2 responses that modulate longevity. It is possible that the PAR-4-dependent activation of AAK-2 extends lifespan and increases stress resistance, while the AAK-2 activation by other upstream kinases only increases resistance to stress. Another possibility would be that a tissue-specific or sub-cellular AAK-2 activation might lead to different outcomes.

Importantly, our data indicate that loss of FLCN-1 extends lifespan only upon treatment with high concentrations (100 uM) of the DNA synthesis inhibitor 5-fluoro-2′-deoxyuridine (FUDR) (Figure S9F and Table S1), a phenotype that has been recently reported by Gharbi et al. [91]. This drug is frequently used in *C. elegans* aging studies to prevent eggs from hatching and has been recently reported to “artifactually” affect lifespan in mitochondrial *C. elegans* mutants and modulate metabolism in the *daf-2* mutant strain [92], [93], [94]. It is not clear why *flcn-1(ok975)* animals exhibit an extension of lifespan only upon treatment with FUDR and what would be the potential mechanism of FUDR action. FUDR may act as metabolic stressor especially that high FUDR concentrations above 100 uM seem to be required to observe the effect on lifespan in *flcn-1(ok975)* animals (Figure S9). Interestingly, lower concentrations of FUDR (5–10 uM) that are also sufficient to inhibit germ line proliferation had no effect on lifespan.

Here we show that the enhanced resistance to oxidative stress in the absence of FLCN-1 does not result from a decrease in oxidative damage or an increased transcriptional upregulation of ROS-detoxifying enzymes [51], [52], [89]. Instead, we show that loss of FLCN-1 activates AAK-2 thereby inducing autophagy. Accordingly, downregulation of *unc-51*, the ortholog of the autophagy kinase ULK1, was shown to suppress the increased number of positive GFP::LGG-1 foci upon overexpression of the kinase domain of AAK-2 in *C. elegans* [46]. More evidence was gathered in mammalian systems to support the direct activation of autophagy by AMPK [46], [47], [48]. In addition, we show that autophagy is required for the increased ATP in *flcn-1(ok975)* animals and *Flcn^−/−^* MEFs suggesting that the chronic activation of autophagy in the absence of FLCN recycles building blocks to produce ATP promoting stress resistance.

When energy levels drop in the cell, AMP or ADP bind to the γ regulatory subunit of AMPK and induce an allosteric conformational change [43], [44], which leads to AMPK activation through phosphorylation of Thr172 in the catalytic subunit via inhibition of dephosphorylation activities. It is striking that loss of FLCN induces AMPK and autophagy in *flcn-1(ok975)* mutant animals and *Flcn^−/−^* MEFs, which exhibit high energy levels. These observations suggest that FLCN might be involved in the control of the energy sensing ability of AMPK. The increased activation of AMPK despite high energy levels has been recently reported upon inhibition of two other inhibitors of AMPK activity [95], [96].

The roles of AMPK and autophagy in cancer are puzzling [97], [98], [99], [100]. Both AMPK activation and autophagy have been shown to acquire anti- and pro-tumor functions [97], [98], [99], [100]. Our results imply that the AMPK-dependent activation of autophagy might be essential for FLCN-deficient tumor cells to acquire an energetic advantage and drive tumorigenesis. In analogy with our results, autophagy was recently shown to be required for tumor growth in many cancer models [101], [102], [103]. A similar role for VHL, another renal tumor suppressor, in the regulation of autophagic events in renal cell carcinomas has been recently described [104]. In fact, the inhibition of autophagy by MiR-204 suppressed the tumor growth in VHL-deficient cells [104]. Moreover, the LC3B/ATG5-dependent autophagy was shown to be required for the development of VHL-deficient renal cell carcinomas in nude mice [104]. We suggest that the AMPK-dependent autophagy activation upon loss of FLCN promotes the survival of transformed cells, which normally undergo severe metabolic stresses as caused by hypoxia and lack of blood vessels [105]. In agreement with our results, four groups have recently reported that AMPK activation drives tumorigenesis via metabolic stress adaptation of different tumors [99], [106], [107], [108]. In fact, AMPK is the best-characterized target of the tumor suppressor LKB1 and it was shown recently that loss of LKB-1/AMPK had a positive effect on tumor initiation but a negative effect on tumor progression/dissemination [98], [109].

The fact that FLCN negatively regulates AMPK strongly implicates that it exerts physiological functions other than being a tumor suppressor. In the past ten years, FLCN was reported to be involved in the regulation of apoptosis, rRNA synthesis, TGF-β signaling, B-cell and stem cell differentiation, ciliogenesis, mitochondrial biogenesis, TOR signaling, epithelial polarization, and cytokinesis without the elucidation of the molecular mechanism [24], [25], [26], [31], [33], [35], [39], [110], [111], [112], [113]. While this report was under review, two reports have shown that loss of folliculin leads to mTOR inhibition and that it is involved in nutrient sensing via acting as a GTPase activating enzyme for the RAG GTPases [114], [115]. It is possible that FLCN acts in two complexes. It binds to RAGs under starvation conditions leading to mTOR inhibition, while in normal conditions FLCN would bind AMPK, inhibiting its activity. However, it is not clear how the reported inhibition of mTOR activity upon loss of FLCN could lead to tumorigenesis, since mTOR was shown to be hyper-activated in tumors of BHD patients and mice devoid of FLCN [25].

In conclusion, we used the model organism *C. elegans* to decipher a genetic pathway, which is regulated by FLCN. We show that FLCN negatively regulates the activity of AMPK, which leads to increased autophagy, energy and survival to metabolic stress. Moreover, we confirmed conservation of this pathway in mammalian cells and suggest that chronic AMPK activation upon loss of FLCN potentiates tumorigenesis via increased autophagy leading to metabolic stress resistance and inhibition of apoptosis, which are two hallmarks of cancer cells [116].

## Materials and Methods

### Antibodies, reagents and plasmids

The FLCN-1 nematode polyclonal antibody was generated in rabbits with a purified GST-FLCN-1 recombinant protein by the McGill animal resource center services. Commercial antibodies and reagents used in this study are listed in Text S1.

### *C. elegans* strains, maintenance, RNAi and lifespan assays

*C. elegans* strains were obtained from the *Caenorhabditis* Genetics Center (see Table S6). The *flcn-1(ok975)* strain was outcrossed eight times to wild-type. Nematodes were maintained and synchronized using standard culture methods [117]. The RNAi feeding experiments were performed as described in [118], and bacteria transformed with empty vector were used as control. For all RNAi experiments, phenotypes were scored with the F1 generation except for *aak-2* knockdown (F2). Lifespan assays were performed according to standard protocols [119].

### Transgenic strain construction

Expression constructs were generated using the pPD95.77 vector. pRF4 rol-6*(su1006)* was used as a co-injector marker. Transgenic lines were generated by microinjection into the gonad of adult hermaphrodite using standard techniques. 2.8 kb endogenous promoter of *flcn-1* was generated by PCR from wild-type genomic DNA (Forward primer 5′AAAACTGCAGCGTCTTCTCGTTTCACAGTAGTCA-3′ and reverse primer: 5′GCTCTAGATTGAATTCTGTAAAAACATGAATTTGA-3′) and cloned into pPD 95.77 at PstI and XbaI sites. *flcn-1* cDNA was obtained from an RT–PCR reaction performed on wild-type animals RNA extracts using the following: forward primer 5′GCTCTAGAATGCAAGCAGTAATAGCACTTTGT-3′ and Reverse primer 5′CGGGATCCACGAGCAGTAGAGGTTTGAGACTG-3′. *flcn-1* cDNA was subsequently cloned into pPD 95.77 (GFP expression plasmid-with *flcn-1* endogenous promoter region) at XbaI and BamHI sites.

### Cell culture

Primary MEFs were isolated from C57BL/6 E12.5 *Flcn* floxed mice. *Ampk^+/+^* and *Ampk^−/−^* MEFs were described in [93]. Cell lines were maintained in Dulbecco's modified Eagle's medium (DMEM) supplemented with 10% fetal bovine serum (FBS), 100 U/ml penicillin and 100 µg/ml streptomycin (Invitrogen). For details on shRNA procedures, transfections, FLCN deletions and immortalization see supporting information.

### Stress resistance assays

Resistance to acute oxidative stress (100 mM PQ and H_2_O_2_) was determined as described in [52], [54]. Chronic oxidative stress was assessed on thirty post-fertile animals using 4 mM PQ and survival was measured daily. For heat stress, one-day adult animals were transferred to NGM plates and exposed to 35°C. Survival was measured at indicated time points until all animals died. Concerning anoxia stress, one-day old adult animals were transferred to NGM plates and left in a Bio-Bag Environmental Chamber Type A (Becton Dickinson Microbiology Systems) for 26 hours at 20°C. Recovery rates were scored at indicated time points. For MEFs, cells were seeded (2×10^4^ cells) in 12-well plates and FBS-free media was added 24 hours after plating. Cell numbers were counted daily and survival rates were determined as the percent cell number compared to day 0.

### Autophagy analysis by fluorescent microscope

For *C. elegans*, autophagy levels were assessed in hypodermal seam cells of L3 animals using the GFP::LGG-1 transgenic reporter strain DA2123 (See Table S6). For MEFs, wild-type and *Flcn^−/−^* cells were infected with the pMigR-1-LC3-GFP or the mcherry/GFP-LC3 constructs [82], seeded on coverslip (50000 cells in 6 well-plate), serum starved for 12 hours and fixed with 4% paraformaldehyde. Autophagic-GFP positive puncta were quantified in at least 200 cells. Pictures from nematodes and MEFs were acquired with a Zeiss fluorescence confocal microscope.

### Determination of ATP content

For *C. elegans*, synchronized young adults were collected and washed in M9 buffer. Pellets were treated with three freeze/thaw cycles and boiled for 15 min. ATP content in *C. elegans* was measured using an ATP determination kit (Invitrogen) and in MEFs using CellTiter-Glo Luminescent Cell Viability Assay (Promega). For *C. elegans*, levels were normalized to protein levels and in MEFs normalized to cell number.

### DNA and protein oxidative damage assays

Genomic DNAs from worm pellets were purified using Phenol/Chloroform extraction and treated with RNase A for 1 hour at 37°C. OxiSelect oxidative DNA damage ELISA assay was performed with 8 µg of DNA following manufacturer's instructions (Cell Biolabs). Protein oxidative damage was assessed using Oxyblot Protein Oxidation Detection Kit (Millipore).

### RNA extraction and real-time PCR

Synchronized young adult nematodes were harvested and total RNA was extracted with Trizol, purified using the RNeasy kit (Qiagen). Quantitative real-time PCR (qRT-PCR) was performed using Express SYBR Green qPCR supermix (Invitrogen) and LightCycler480 system (Roche). Catalase and SOD transcripts were normalized to housekeeping genes *cdc42*, *pmp-3*, and *Y45F10D.4* using Genorm [120]. AAK transcripts were normalized against *cdc-42*. For primer sequences see Table S7.

### Protein extraction and western blotting

Cells and synchronized young adult nematodes were washed with ice-cold PBS and M9 respectively and lysed in the AMPK lysis buffer [121] supplemented with the complete protease and phosphatase inhibitors (Roche), 1 mM DTT, and benzamidine 1 µg/ml. Worm pellets were sonicated and cleared by centrifugation. Percent pAAK-2/pAMPK levels were quantified using ImageJ software and normalized for the AMPK levels.

### Transmission electron microscopy

Synchronized young adult nematodes were treated with 50 mM PQ/M9 or M9 alone for 2 hours, washed and plated on NGM plates allowing 30 minutes recovery. TEM immersion fixation and embedding was performed according to [122]. See Text S1.

### Soft agar assay

FTC cells were trypsinized, counted and resuspended in complete DMEM/F12 media. Two layered soft agar assay were undertaken in six well plates. The bottom layer contains 0.6% agar in complete DMEM/F12 media. The second layer encompasses 0.3% agar mixed with 0.5 million cells. Plates were cultured at 37°C in 5% CO2.

### Apoptosis assay

For worms, apoptotic germ cell corpses were visualized using Acridine Orange (AO) as described in [123]. Worms were incubated for 2 hours in M9 with or without 50 mM PQ in OP50, supplemented with 2 µl/ml AO (stock of 10 mg/ml). Worms were then washed and transferred into light-protected recovery NGM plates for 45 min before visualization. In MEFs, apoptosis levels were determined using the Annexin V: PE Apoptosis Detection Kit I (BD Pharmingen) according to manufacturer's protocol. Fluorescence intensity corresponding to apoptosis levels was detected using FACSCalibur flow cytometer (excitation 488; emission 575/26; BD Biosciences).

### LC-MS/MS analysis

Targeted metabolite analysis was performed on an Agilent 6430 triple quadrupole mass spectrometer equipped with a 1290 Infinity UPLC system (Agilent Technologies). Metabolites were separated using a 4.0 µm, 2.1×100 mm Cogent Diamond Hydride column (MicroSolv Technology Corporation). Quantification was accomplished using MassHunter Quantitative Analysis software (Agilent). See Text S1 for details.

### Statistical analyses

Data are expressed as means ±SEM. Statistical analyses for all data were performed by unpaired student's t-test, ANOVA, using Excel (Microsoft, Albuquerque, NM, USA), SPSS (IBM, Armonk, NY, USA) and prism software (GraphPad). For lifespan curve comparisons we used the Log-rank Mantel Cox test using GraphPad from Prism Statistical significance is indicated in figures (* *P*<0.05, **P<0.01, ***P<0.001) or included in the supplementary tables.

## Supporting Information

Figure S1 Loss of FLCN promotes AMPK-dependent oxidative stress resistance in *C. elegans*. (A) Survival of wild-type and *flcn-1(ok975)* animals after 3 hours of treatment with increasing H_2_O_2_ concentrations. (B) Relative mRNA levels of the indicated *C. elegans* AAK-1 and AAK-2 genes in wild-type and *flcn-1(ok975)* animals. Levels were normalized to *cdc-42*. Data represent the mean ± SEM, n≥3.
(EPS)

Figure S2 The increased survival of *flcn-1* animals to paraquat might not be dependent on classical oxidative stress resistance mechanisms. (A and B) Percent survival of indicated worm strains treated with 100 mM PQ. See also Table S3 (C) Relative mRNA levels of the indicated *C. elegans* catalase and SOD genes in wild-type and *flcn-1(ok975)* animals. (D and E) Quantification of the oxidative protein (D) and DNA damage (E) measured in animals treated with or without PQ. Data represent the mean ± SEM, n≥3.
(EPS)

Figure S3 Expression levels of *lgg-1*. (A) Relative mRNA levels of *C. elegans lgg-1* in (A) GFP::LGG-1 and *flcn-1*;GFP::LGG-1 strains (n≥2) and (B) wild-type and *flcn-1(ok975)* animals (n≥3).
(EPS)

Figure S4 Autophagy activation conferred by loss of Folliculin protects against apoptosis. (A) Quantification of the number of acridine orange positive apoptotic cell corpses in the gonad arms of the indicated strains. (B–E) Percent survival of the indicated worm strains treated with the indicated RNAi feeding clones and exposed to 100 mM PQ. See also table S3. Data represent the means ± SEM N≥3.
(EPS)

Figure S5 Loss of FLCN promotes AMPK-dependent oxidative stress resistance in MEFs. (A) Percent survival of the indicated MEFs treated with 2 mM H_2_O_2_. (B) Western blot analysis of pAMPK (Thr172) and AMPK protein levels in indicated MEFs lines. (C) Western blot analysis performed on protein cell lysates using the indicated antibodies. MEFs were serum starved for 24 hours or treated with 0.5 mM AICAR for 15 hours.
(EPS)

Figure S6 Loss of FLCN induces autophagy in MEFs. (A) Representative immunofluorescence pictures of LC3 positive mCherry and GFP puncta under normal conditions or upon treatment with 10 µM CQ. Arrows indicate autophagosomes. (B) Immunoblot analysis of LC3 cleavage profile in the indicated MEFs lines under normal conditions or upon serum starvation. (C) Percent survival of indicated cell lines upon serum starvation, treated with or without *Beclin-1* shRNA. Data represent the mean ± SEM, n≥3.
(EPS)

Figure S7 Loss of FLCN stimulates cellular energy production and availability. Bioenergetic metabolites (A), relative energy charge (B) and phospho-creatine (C) levels, determined by LC-MS analysis in the indicated MEFs incubated with or without serum-free media for 24 hours. (D) Apoptosis levels in indicated cell lines upon serum starvation treated with or without CQ. Data represent the mean ± SD, n≥3.
(EPS)

Figure S8 Loss of FLCN in tumor cells confers higher cellular energy levels dependent on increased autophagy. (A) Western blot analysis of LC3 and AMPK phosphorylation levels on Threonine 172 (pAMPK) revealed in the indicated FTC cell lines. Folliculin (FLCN) and actin expression levels are shown as controls. (B) Western blot analysis of ATG7 in the indicated FTC cell lines. Tubulin expression level is shown as control. The percent downregulation is quantified using ImageJ software and is normalized for the tubulin levels. Data represent the means of at least three independent experiments. Relative ATP levels measured in the indicated FTC cell lines. Data represent the means ± S.D. (C) Soft agar colonies assays in the indicated FTC cell lines. (D) Quantification relative to control in the indicated FTC cell lines. Data represent the mean ± S.D.
(EPS)

Figure S9 High FUDR concentrations are required to observe the increase in lifespan in *flcn-1(ok975)* animals compared to wild-type. Representative lifespan survival curves of wild-type and *flcn-1(ok975)* animals exposed to 0 µ (A), 1 µ (B), 5 µM (C), 10 µM (D), 50 µM (E), 100 µM (F), and 150 µM (G) of FUDR. P values are calculated according to the Log-rank Mantel Cox test.
(EPS)

Table S1 Lifespan results and statistical analysis.
(DOCX)

Table S2 Percent survival upon mild oxidative stress: results and statistical analysis.
(DOCX)

Table S3 Percent survival upon acute oxidative stress: results and statistical analysis.
(DOCX)

Table S4 Mean survival to heat stress: results and statistical analysis.
(DOCX)

Table S5 Percent recovery (1 hour) after anoxic stress: results and statistical analysis.
(DOCX)

Table S6 Strain list.
(DOCX)

Table S7 Primer sequences.
(DOCX)

Text S1 Supplementary Material and Methods.
(DOCX)
