# Supplementary material for: Folliculin Regulates Ampk-Dependent Autophagy and Metabolic Stress Survival
Source: PLoS Genet. 2014 Apr 24;10(4):e1004273. doi: 10.1371/journal.pgen.1004273 (PMC3998892; doi:10.1371/journal.pgen.1004273)
Supplement: Table S1 — Lifespan results and statistical analysis. (DOCX) [file pgen.1004273.s010.docx]

| **Table S1. Lifespan results and statistical analysis** | | | | |
| --- | --- | --- | --- | --- |
| Strain | Mean survival  (days±SEM) | p-value | Number of Experiments(n) | Number of Nematodes(n) |
| N2 | 14.20 ± 0.3 |  | 5 | 384 |
| *flcn-1(ok975)* | 14.80 ± 0.5 | n.s.^a^ | 5 | 378 |
| N2 (100µM FUDR) | 16.28 ± 0.9 |  | 5 | 273 |
| *flcn-1(ok975)*(100µM FUDR) | 21.01 ± 0.7 | <0.0001^b^ | 5 | 302 |

1. Compared to N2 animals grown on NGM plates
2. Compared to N2 animals grown on NGM plates supplemented with FUDR.
