# Supplementary material for: Folliculin Regulates Ampk-Dependent Autophagy and Metabolic Stress Survival
Source: PLoS Genet. 2014 Apr 24;10(4):e1004273. doi: 10.1371/journal.pgen.1004273 (PMC3998892; doi:10.1371/journal.pgen.1004273)
Supplement: Table S2 — Percent survival upon mild oxidative stress: results and statistical analysis. (DOCX) [file pgen.1004273.s011.docx]

| **Table S2. Percent survival upon mild oxidative stress (4mM): results and statistical analysis** | | | | |
| --- | --- | --- | --- | --- |
| Strain, RNAi, treatment | Mean survival  (days±SEM) | p-value | Number of Experiments(n) | Number of Nematodes(n) |
| N2-PQ | 3.2 ± 0.06 |  | 3 | 193 |
| *flcn-1(ok975)-*PQ | 4.6 ± 0.3 | <0.0001^a^ | 3 | 116 |
| *flcn-1(ok975); flcn-1::GFP line1-*PQ | 3.4 ± 0.2 | <0.05^b^  /n.s.^a^ | 3 | 102 |
| *flcn-1(ok975); flcn-1::GFP line2-*PQ | 3.1 ± 0.03 | <0.05^b^  /n.s.^a^ | 3 | 88 |
| N2-NT (non treated) | 7.2 ± 0.3 | <0.0001^a^ | 5 | 384 |
| *flcn-1(ok975)-*NT (non treated) | 7.8 ± 0.5 | <0.0001^b^ | 5 | 378 |
| N2-PQ | 3.1 ± 0.07 |  | 3 | 92 |
| *flcn-1(ok975)-* PQ | 4.3 ± 0.32 | <0.0001^c^ | 3 | 88 |
| *aak-2(ok524)-* PQ | 1.2 ± 0.06 | <0.0001^c^ | 3 | 80 |
| *aak-2(ok524); flcn-1 (ok975)-*PQ | 1.2 ± 0.03 | <0.0001^d^  /n.s.^e^ | 3 | 81 |
| *aak-2(ok524)*-NT | 3.8+0 | <0.0001 ^e^ | 1 | 126 |
| *aak-2(ok524)*; *flcn-1 (ok975)*-NT | 3.5+0 | <0.0001 ^f^ | 1 | 89 |
| N2 (control RNAi)-PQ | 3.0 ± 0.16 |  | 3 | 96 |
| *flcn-1(ok975)* (control RNAi)-PQ | 4.3 ± 0.40 | <0.0001^g^ | 3 | 113 |
| N2 (*bec-1*RNAi)-PQ | 1.1 ± 1.14 | <0.0001^g^ | 3 | 94 |
| *flcn-1(ok975)*(*bec-1* RNAi*)-*PQ | 1.5 ± 0.12 | <0.0001^h^ /n.s.^i^ | 3 | 81 |
| N2 (control RNAi)-NT | 7.4± 0.44 | <0.0001^g^ | 3 | 266 |
| *flcn-1(ok975)* (control RNAi)-NT | 5.8± 0.06 | <0.05 ^h^ | 3 | 193 |
| N2 (*bec-1*RNAi)-NT | 4.7 ± 0.37 | <0.0001^i^ | 3 | 307 |
| *flcn-1(ok975)*( *bec-1* RNAi)-NT | 3.9 ± 0.78 | <0.05 ^j^ | 3 | 313 |
| N2 (control RNAi)-PQ | 4.1 ± 0.18 |  | 3 | 87 |
| *flcn-1(ok975)* (control RNAi)-PQ | 5.5 ± 0.23 | <0.0001^k^ | 3 | 97 |
| N2 (*atg-7* RNAi)-PQ | 3.0 ± 0.15 | <0.0001^k^ | 3 | 84 |
| *flcn-1(ok975)*(*atg-7* RNAi)-PQ | 2.9 ±0.22 | <0.0001^l^  /n.s.^m^ | 3 | 80 |
| N2 (control RNAi)-NT | 7.4± 0.44 | <0.0001^k^ | 3 | 266 |
| *flcn-1(ok975)* (control RNAi)-NT | 5.8± 0.06 | <0.0001^l^ | 3 | 193 |
| N2 (*atg-7* RNAi) NT | 6.6 ± 0.38 | <0.0001^m^ | 3 | 266 |
| *flcn-1(ok975)*(*atg-7* RNAi) NT | 5.9 ±0.32 | <0.0001^n^ | 3 | 272 |
| N2 (control RNAi)-PQ | 3.5 ± 0.22 |  | 3 | 97 |
| *flcn-1(ok975)* (control RNAi)-PQ | 5.7 ± 0.43 | <0.0001^o^ | 3 | 102 |
| N2*(flcn-1* RNAi*)-*PQ | 5.0 ± 0.30 | <0.0001^o^  /n.s^p^ | 3 | 86 |
| *flcn-1(ok975)*(*flcn-1* RNAi*)-*PQ | 5.2 ± 0.45 | <0.0001^o^  /n.s.^p^/n.s.^q^ | 3 | 88 |

1. Compared to N2 animals treated with 4mM PQ
2. Compared to *flcn-1(ok975)* animals treated with 4mM PQ
3. Compared to N2 animals treated with 4mM PQ
4. Compared to *flcn-1(ok975)* animals treated with 4mM PQ
5. Compared to *aak-2(ok524)* animals treated with 4mM PQ
6. Compared to *flcn-1(ok975);aak-2(ok524)* animals treated with 4mM PQ.
7. Compared to N2 animals treated with control RNAi and 4mM PQ.
8. Compared to *flcn-1(ok975)* animals treated with control RNAi and 4mM PQ.
9. Compared to N2 animals treated with *bec-1* RNAi and 4mM PQ.
10. Compared to *flcn-1(ok975)* animals treated with *bec-1* RNAi and 4mM PQ.
11. Compared to N2 animals treated with control RNAi and 4mM PQ.
12. Compared to *flcn-1(ok975)* animals treated with control RNAi and 4mM PQ.
13. Compared to N2 animals treated with *atg-7* RNAi and 4mM PQ.
14. Compared to *flcn-1(ok975)* animals treated with *atg-7* RNAi and 4mM PQ.
15. Compared to N2 animals treated with control RNAi and 4mM PQ
16. Compared to *flcn-1(ok975)* animals treated with control RNAi and 4mM PQ
17. Compared to N2 animals treated with *flcn-1* RNAi and 4mM PQ
