# Supplementary material for: Folliculin Regulates Ampk-Dependent Autophagy and Metabolic Stress Survival
Source: PLoS Genet. 2014 Apr 24;10(4):e1004273. doi: 10.1371/journal.pgen.1004273 (PMC3998892; doi:10.1371/journal.pgen.1004273)
Supplement: Table S3 — Percent survival upon acute oxidative stress: results and statistical analysis. (DOCX) [file pgen.1004273.s012.docx]

| **Table S3.Percent survival upon acute oxidative stress (100mM): results and statistical analysis** | | | | |
| --- | --- | --- | --- | --- |
| Strain, RNAi | Percent Survival (4h) (±SEM) | p-value | Number of experiments  (n) | Number of worms (n) |
| N2 | 40.06 ± 3.29 |  | 3 | 94 |
| *flcn-1(ok975)* | 64.64 ± 0.47 | <0.01 ^a^ | 3 | 175 |
| *flcn-1(ok975); flcn-1::GFP line1* | 42.67 ± 8.28 | <0.05 ^b^ / n.s.^a^ | 3 | 106 |
| *flcn-1(ok975); flcn-1::GFP line2* | 41.03 ± 8.9 | <0.05 ^b^ / n.s.^a^ | 3 | 152 |
| N2 | 35.08 ± 3.36 |  | 4 | 235 |
| *flcn-1(ok975)* | 53.00 ± 3.32 | <0.05 ^c^ | 4 | 227 |
| *aak-2(ok524)* | 22.57 ± 2.22 | <0.01 ^c^  <0.001 ^d^ | 4 | 203 |
| *flcn-1(ok975);aak-2(ok524)* | 21.68 ± 4.23 | < 0.01 ^d^  /n.s. ^e^ | 4 | 215 |
| N2 | 46.13 ± 7.83 |  | 3 | 167 |
| *flcn-1(ok975)* | 66.00 ± 1.37 | <0.05 ^f^ | 3 | 163 |
| *aak-1(tm144)* | 46.91 ± 7.38 | n.s. ^f^ | 3 | 183 |
| *flcn-1(ok975);aak1(tm1944)* | 61.71 ± 3.58 | n.s. ^g^ / <0.05 ^h^ | 3 | 203 |
| N2 | 25.78 ± 6.72 |  |  | 163 |
| *flcn-1(ok975)* | 50.58 ± 9.82 | <0.05 ^i^ | 3 | 173 |
| *par-4(it57)* | 20.54 ± 0.095 |  | 3 | 167 |
| *flcn-1(ok975); par-4(it57)* | 35.03 ± 4.365 | <0.01 ^J^ | 3 | 177 |
| N2 | 30.31 ± 2.44 |  | 3 | 169 |
| *flcn-1(ok975)* | 50.17 ± 2.67 | <0.05 ^k^ | 3 | 134 |
| *daf-2(e1370)* | 59.26 ± 0.1 | <0.05 ^k^ | 3 | 177 |
| *flcn-1(ok975);daf-2(e1370)* | 75.43 ± 0.43 | <0.05 ^l^ | 3 | 162 |
| N2 | 40.90 ± 9.76 |  | 3 | 184 |
| *flcn-1(ok975)* | 54.71 ± 8.89 | <0.01 ^m^ | 3 | 193 |
| *daf-16(mu86)* | 28.62 ± 6.96 | <0.05 ^m^ | 3 | 188 |
| *daf-16(mu86); flcn-1(ok975)* | 50.00 ± 5.92 | <0.01 ^n^ | 3 | 193 |
| N2 (control RNAi) | 28.53 ± 8.55 |  | 5 | 292 |
| *flcn-1 (ok975)* (control RNAi) | 56.63 ± 8.89 | <0.05 ^o^ | 5 | 269 |
| N2 (*atg-7* RNAi) | 33.97 ± 5.14 |  | 5 | 294 |
| *flcn-1(ok975)*(*atg-7* RNAi) | 30.70 ± 10 | <0.05 ^p^ / n.s. ^q^ | 5 | 280 |
| N2 (control RNAi) | 28.53 ± 8.55 |  | 3 | 292 |
| *flcn-1(ok975)* (control RNAi) | 56.63 ± 8.89 | <0.05 ^o^ | 3 | 269 |
| N2 (*bec-1*RNAi) | 26.58 ± 8.40 |  | 3 | 272 |
| *flcn-1(ok975)*(*bec-1*RNAi) | 26.62 ± 4.70 | <0.05 ^p^ / n.s. ^r^ | 3 | 257 |
| N2 (control RNAi) | 47.17 ± 7.07 |  | 5 | 300 |
| *flcn-1(ok975)* (control *RNAi*) | 66.15 ± 4.23 | <0.001 ^s^ | 5 | 339 |
| N2 *(ced-3* RNAi*)* | 67.36 ± 5.18 | <0.01 ^s^ | 5 | 339 |
| *flcn-1(ok975)*(*ced-3* RNAi*)* | 70.39 ± 5.20 | n.s. ^t^ | 5 | 324 |
| N2 (control RNAi) | 52.52 ± 1.67 |  | 3 | 173 |
| *flcn-1(ok975)* (control RNAi) | 71.06 ± 3.73 | <0.01 ^u^ | 3 | 223 |
| N2 (*ced-9* RNAi) | 49.07 ± 2.44 | n.s. ^u^ | 3 | 170 |
| *flcn-1(ok975)*(*ced-9* RNAi) | 57.62 ± 4.72 | <0.05 ^v^ / n.s. ^w^ | 3 | 184 |
| N2 (control RNAi) | 47.51 ± 6.42 |  | 3 | 196 |
| *flcn-1(ok975)* (control RNAi) | 67.12 ± 4.51 | <0.05 ^x^ | 3 | 225 |
| N2 (*egl-1* RNAi) | 68.50 ± 1.22 | <0.05 ^x^/ n.s. ^y^ | 3 | 213 |
| *flcn-1(ok975)*(*egl-1* RNAi) | 70.28 ± 7.53 | n.s. ^z^ / n.s. ^y^ | 3 | 218 |
| N2 (control RNAi) | 52.52 ± 7.23 |  | 3 | 203 |
| N2 (*ced-3* RNAi) | 74.96 ± 1.69 | <0.05 ^aa^ | 3 | 171 |
| *aak-2(ok 524)* (control RNAi) | 55.09 ± 10.61 |  | 3 | 165 |
| *aak-2(ok 524)*(*ced-3* RNAi) | 54.33 ± 7.76 | n.s. ^bb^ | 3 | 216 |
| N2 (control RNAi) | 51.21 ± 2.25 |  | 3 | 202 |
| *flcn-1(ok975)* (control RNAi) | 70.50 ± 9.15 | <0.05 ^cc^ | 3 | 207 |
| N2 (*let-363* RNAi) | 63.73 ± 1.53 | <0.01 ^cc^ | 3 | 187 |
| *flcn-1(ok975)*(*let-363* RNAi) | 83.18 ± 3.42 | <0.01 ^dd^ | 3 | 221 |

1. Compared to *N2* animals.
2. Compared to *flcn-1*(ok975) animals.
3. Compared to N2 animals.
4. Compared to *flcn-1(ok975)* animals.
5. Compared to *aak-2(ok524)* animals.
6. Compared to N2 animals.
7. Compared to *flcn-1(ok975)* animals.
8. Compared to *aak-1(tm1944)* animals.
9. Compared to N2 animals.
10. Compared to *par-4(it57)* animals.
11. Compared to N2 animals.
12. Compared to *daf-2(e1370)* animals.
13. Compared to N2 animals.
14. Compared to *daf-16(mu86)* animals.
15. Compared to N2 animals.
16. Compared to *flcn-1(ok975)* animals treated with control RNAi.
17. Compared to N2 animals treated with *atg-7* RNAi.
18. Compared to N2 animals treated with *bec-1* RNAi.
19. Compared to N2 animals treated with control RNAi.
20. Compared to N2 animals treated with *ced-3* RNAi.
21. Compared to N2 animals treated with control RNAi.
22. Compared to *flcn-1(ok975)* treated with control RNAi.
23. Compared to N2 animals treated with *ced-9* RNAi.
24. Compared to N2 animals treated with control RNAi.
25. Compared to *flcn-1(ok975)* animals treated with control RNAi.
26. Compared to N2 animals treated with *egl-1* RNAi
27. Compared to N2 animals treated with control RNAi.
28. Compared to *aak-2(ok524)* animals treated with control RNAi.
29. Compared to N2 animals treated with control RNAi.
30. Compared to N2 animals treated with *let-363* RNAi.
