# Supplementary material for: Folliculin Regulates Ampk-Dependent Autophagy and Metabolic Stress Survival
Source: PLoS Genet. 2014 Apr 24;10(4):e1004273. doi: 10.1371/journal.pgen.1004273 (PMC3998892; doi:10.1371/journal.pgen.1004273)
Supplement: Table S4 — Mean survival to heat stress: results and statistical analysis. (DOCX) [file pgen.1004273.s013.docx]

| **Table S4. Mean survival to heat stress: results and statistical analysis** | | | | |
| --- | --- | --- | --- | --- |
| Strain | Mean survival  (hours±SEM) | p-value | Number of experiments  (n) | Number of worms(n) |
| N2 | 7.09 ± 0.39 |  | 4 | 312 |
| *flcn-1(ok975)* | 9.51 ± 0.47 | <0.001 | 4 | 261 |
