# Supplementary material for: Folliculin Regulates Ampk-Dependent Autophagy and Metabolic Stress Survival
Source: PLoS Genet. 2014 Apr 24;10(4):e1004273. doi: 10.1371/journal.pgen.1004273 (PMC3998892; doi:10.1371/journal.pgen.1004273)
Supplement: Table S5 — Percent recovery (1 hour) after anoxic stress: results and statistical analysis. (DOCX) [file pgen.1004273.s014.docx]

| **Table S5. Percent recovery (1 hour) after anoxic stress: results and statistical analysis** | | | | |
| --- | --- | --- | --- | --- |
| Strain | Percent recovery  (±SEM) | p-value | Number of Experiments  (n) | Number of worms  (n) |
| N2 | 35.04 ± 7.42 |  | 4 | 154 |
| *flcn-1(ok975)* | 81.40 ± 8.12 | <0.01 | 4 | 143 |
