# Supplementary material for: Folliculin Regulates Ampk-Dependent Autophagy and Metabolic Stress Survival
Source: PLoS Genet. 2014 Apr 24;10(4):e1004273. doi: 10.1371/journal.pgen.1004273 (PMC3998892; doi:10.1371/journal.pgen.1004273)
Supplement: Table S6 — Strain list. (DOCX) [file pgen.1004273.s015.docx]

| **Table S6. Strain list** | | |
| --- | --- | --- |
| Genotype | Strain Number | Additional information |
| wild-type Bristol (N2) |  |  |
| *flcn-1(ok975) II* |  | RB1035 strain was outcrossed 8 times with wild-type Bristol (N2) |
| *aak-1(tm1944) III* | FX1944 | from CGC |
| *aak-2(ok524) X* | RB754 | from CGC |
| *daf-16(mu86) I* | CF1038 | from CGC |
| *daf-2(e1370) III* | CB1370 | from CGC |
| *adIs2122[lgg-1::GFP + rol-6(su1006)]* | DA2123 | from CGC |
| *par-4(it57)V* | KK184 | from CGC |
| *flcn-1(ok975); flcn::GFP(1)* |  | Overexpression of FLCN-1 co-injected with *rol-6(su1006)gf* |
| *flcn-1(ok975); flcn::GFP(2)* |  | Overexpression of FLCN-1 co-injected with *rol-6(su1006)gf* |
| *flcn-1(ok975) II; aak-2(ok524) X* |  |  |
| *flcn-1(ok975) II; par-4(it54) V* |  |  |
| *flcn-1(ok975) II; daf-2(e1370) III* |  |  |
| *daf-16(mu86) I; flcn-1(ok975) II* |  |  |
