# Supplementary material for: Folliculin Regulates Ampk-Dependent Autophagy and Metabolic Stress Survival
Source: PLoS Genet. 2014 Apr 24;10(4):e1004273. doi: 10.1371/journal.pgen.1004273 (PMC3998892; doi:10.1371/journal.pgen.1004273)
Supplement: Table S7 — Primer sequences. (DOCX) [file pgen.1004273.s016.docx]

| **Table S7. Primer sequences** | | |
| --- | --- | --- |
| Gene symbol | Forward primer | Reverse primer |
| *ctl-1* | GCGGATACCGTACTCGTGAT | AATCCGGATGAACTTTCGTG |
| *ctl-2* | CATCCGTGACCTGTTCAATG | TGGATTTCTGTTCAGCACCA |
| *ctl-3* | CTGGGAGAAAGTGCTCAAGG | TTCTTCTGGCAGAGCTGATG |
| *sod-1* | ACGCTTTACGGTCCAAACAC | CTTCTGCCTTGTCTCCGACT |
| *sod-2* | TGGACTTGTTCCACTGTTCG | CTCGCTGACGTTCTTCCAGT |
| *sod-3* | CTATTGCGGTTCAAGGCTCT | TGGCAAATCTCTCGCTGATA |
| *sod-4* | TCCGACTCTCTTGCCTCATT | AGGGATGCTGTCGTTGTTTC |
| *sod-5* | ACGTTGGTGACCTTGGAAAC | GAGCAATGACTCCACAAGCA |
| *cdc-42* | GGCAAAGGAATTGAAAGCAG | GGGGGCTAAGAAAATTTGGA |
| *pmp-3* | GTTCCCGTGTTCATCACTCAT | ACACCGTCGAGAAGCTGTAGA |
| *y45f10d.4* | GTCGCTTCAAATCAGTTCAGC | GTTCTTGTCAAGTGATCCGACA |
| *aak-1* | TTGGAAAAGGAGCATTTGGA | AACACGGAAGAGACGTGTGA |
| *aak-2* | ATAGGAAGGAGGACGGTGGT | CTTCGTCGACGTTTCTCCTC |
| *lgg-1* | GAAAACGCATCCAACTTCGT | TCGGCGGATAATACATGACA |
