# Supplementary material for: Folliculin Regulates Ampk-Dependent Autophagy and Metabolic Stress Survival
Source: PLoS Genet. 2014 Apr 24;10(4):e1004273. doi: 10.1371/journal.pgen.1004273 (PMC3998892; doi:10.1371/journal.pgen.1004273)
Supplement: Text S1 — Supplementary Material and Methods. (DOCX) [file pgen.1004273.s017.docx]

**Supplementary Material and Methods**

**Antibodies and reagents**

### The ULK1-pSer555 (5869), AMPK-α (2532), AMPK-pThr172 (2531), ACC (3676), ACC-pSer79 (3661) and LC3B (2775) were purchased from Cell Signaling and the β-actin (AC74) antibody from Sigma-Aldrich. The following reagents were purchased from Sigma-Aldrich: AICAR, methyl viologen dichloride hydrate (Paraquat; PQ), chloroquine (CQ), Acridine Orange.

**Electron microscopy**

Transmission electron microscopy procedures were performed according to Hall et al., 1995 [122]. Briefly, synchronized L4 wild-type and *flcn-1* animals were separated in two groups and treated with M9 buffer or 50mM paraquat in M9 solution for 2 hours. After incubation, animals were washed three times with M9 buffer and plated on NGM plates allowing 30 minutes recovery. Animals were fixed at room temperature in 1% formaldehyde, 2.5% glutaraldehyde in 0.12 M sodium cacodylate buffer, at pH 7.4. Under a dissecting microscope, animals were cut open in a drop of the fix solution, allowing the fixative to penetrate. After 2 hours, animals were washed three times with 0.15M sodium-cacodylate buffer, at pH7.4. Animals were then post-fixed on ice for one hour in 1% Osmium tetroxide, 0.5% potassium ferrocyanide in 0.12M sodium-cacodylate buffer, at pH 7.4. Following post-fixation, animals were rinsed three times with 0.15M sodium cacodylate (pH7.4) and three more times with 0.15 sodium acetate buffer (pH5.2), and then stained for 7 hours at 4⁰C, with 1% uranyl acetate in 1M sodium acetate buffer (pH5.2). Animals were then washed multiple times with 0.15M sodium acetate buffer and double distilled water, grouped, and embedded in 2% agarose in double distilled water. Agarose blocks were dehydrated with a series of increasing ethanol concentrations (30%, 50%, 70%, 95%, and 100% ethanol) and then placed for 45 min in 100% propylene oxide. Blocks were gradually infiltrated with resin using 1:3, 1:1, and 3:1 resin/propylene oxide mixtures. Blocks were further incubated in 100% resin for several hours, then positioned in a flat embedding mold and incubated in the oven at 60⁰C for more than 2 days. Thin sections were cut on an RMC Powertome XL (Boeckler Instruments) using a diamond knife (DDK) and collected on Pioloform-coated copper slot grids. Grids were post-stained with 4% uranyl acetate and lead citrate and viewed using a Philips CM10 electron microscope (FEI) equipped with a Morada digital camera (Olympus) and iTEM software (Olympus SIS). For MEFs same protocol was used except that thecells were plated in Lab-Tek chamber Slide, washed with 0.1M sodium Cacodylate three times the day after and fixed 2% paraformaldehyde 2.5% glutaraldehyde in cacodylate buffer.

**LC-MS**

### Targeted metabolite analysis was performed on an Agilent 6430 triple quadrupole mass spectrometer equipped with a 1290 Infinity UPLC system (Agilent Technologies, Santa Clara, CA, USA). Sample temperature was maintained at 4°C while solvents and column temperatures were maintained at 10°C. Metabolites were separated using a 4.0μm, 2.1×100.0mm Cogent Diamond Hydride column (MicroSolv Technology Corporation, Eatontown, NJ, USA) operating at a flow rate of 0.4 ml/min and 5 μl sample injections. Separation solvent “A” consisted of 15 mM ammonium formate in H_2_O, pH 5.8 and solvent “B” consisted of 15 mM ammonium formate in 85% acetonitrile in 15% H_2_O, pH 5.8. Chromatography started with a 2 min hold at 97% B, followed by a 5 min gradient to 70% B, then washed for 3 min with 98% A, and re-equilibrated to starting conditions for 6 min. Separated metabolites were introduced to the mass spectrometer via electrospray ionization (ESI) operating in either positive or negative ionization mode and were analyzed by previously optimized multiple reaction monitoring (MRM). Quantification was accomplished by comparing MRM peak areas to those from standard calibration curves using MassHunter Quantitive Analysis software (Agilent, Santa Clara, CA, USA). MRM transitions in negative ionization mode for quantifying and qualifying ions were 506.0 → 158.9 and 506.0 → 78.9 for ATP, 426.0 → 134 and 426.0 → 79 for ADP, 346.0 → 97 and 346.0 → 78.9 for AMP, 179.0 → 89.0 and 179 → 59 for glucose and 89 → 43.1 for lactate. MRM transitions in positive ionization mode were 212.0 → 90.1 and 212.0 → 114.1 for phosphocreatine and 132.0 → 44.2 and 132.0 → 90.1 for creatine. Gas temperature and flow were set at 350°C and 10 L/min. Nebulizer pressure was set at 50 psi and capillary voltage was +4000V.
